# Gut microbiota profiles in diarrheic patients with co-occurrence of *Clostridioides difficile* and *Blastocystis*

**DOI:** 10.1371/journal.pone.0248185

**Published:** 2021-03-16

**Authors:** Laura Vega, Giovanny Herrera, Marina Muñoz, Manuel A. Patarroyo, Jenny G. Maloney, Monica Santín, Juan David Ramírez

**Affiliations:** 1 Centro de Investigaciones en Microbiología y Biotecnología-UR (CIMBIUR), Facultad de Ciencias Naturales, Universidad del Rosario, Bogotá, Colombia; 2 Molecular Biology and Immunology Department, Fundación Instituto de Inmunología de Colombia (FIDIC), Bogotá, Colombia; 3 Microbiology Department, Faculty of Medicine, Universidad Nacional de Colombia, Bogotá, Colombia; 4 USDA ARS, Environmental Microbial and Food Safety Laboratory, BARC, Beltsville, MD, United States of America; Washington State University - Spokane, UNITED STATES

## Abstract

*Blastocystis* and *Clostridioides difficile* co-occurrence is considered a rare event since the colonization by *Blastocystis* is prevented under a decrease in beneficial bacteria in the microbiota when there is *C*. *difficile* infection (CDI). This scenario has been reported once, but no information on the gut microbiota profiling is available. The present study is motivated by knowing which members of the microbiota can be found in this rare scenario and how this co-occurrence may impact the abundance of other bacteria, eukaryotes or archaea present in the gut microbiota. This study aimed to describe the bacterial and eukaryotic communities using amplicon-based sequencing of the 16S- and 18S-rRNA regions of three patient groups: (1) *Blastocystis* and *C*. *difficile* infection (B+/C+, n = 31), (2) *C*. *difficile* infection only (B˗/C+, n = 44), and (3) without *Blastocystis* or *C*. *difficile* (B˗/C˗, n = 40). *Blastocystis* was subtyped using amplicon-based sequencing of the 18S-rRNA gene, revealing circulation of subtypes ST1 (43.4%), ST3 (35.85%) and ST5 (20.75%) among the study population. We found that B+/C+ patients had a higher abundance of some beneficial bacteria (such as butyrate producers or bacteria with anti-inflammatory properties) compared with non-*Blastocystis*-colonized patients, which may suggest a shift towards an increase in beneficial bacteria when *Blastocystis* colonizes patients with CDI. Regarding eukaryotic communities, statistical differences in the abundance of some eukaryotic genera between the study groups were not observed. Thus, this study provides preliminary descriptive information of a potential microbiota profiling of differential presence by *Blastocystis* and *C*. *difficile*.

## Introduction

The gut microbiota is defined as the assembly or microorganisms from different domains (bacteria, archaea, eukaryotes and viruses) that can have a well-defined habitat in the intestine, where their microbial structural elements and the surrounding environmental conditions are considered (microbiome) [1]. Several exogenous and endogenous factors that can generate variations in the composition of the microbiome have been described and this has led to the intestinal microbiota being viewed as a potential indicator of host health [2, 3]. Because multiple factors can alter the composition of the microbiome, it can be difficult to establish a direct cause and effect. To date, most studies on the microbiota have focused on the analysis of different groups of bacteria and thus our understanding of the roles and interactions of eukaryotes, archaea and viruses within the microbiome remains limited [2, 4, 5].

In 2016, Gorvitovskaia *et al*. proposed that certain bacterial taxa can be used as biomarkers since they correlated with diet or a state of disease [6]. Also, in the case of eukaryotes a study proposed a set of protists that can be commonly found in the microbiota: Common luminal intestinal parasitic protists (CLIPPs). CLIPPs are protists (*Blastocystis*, *Giardia*, *Cryptosporidium*, *Dientamoeba*, *Endolimax*) that can be found colonizing gut microbiota, and they appear to be more common than previously thought. It has been suggested that some CLIPPs may have pathogenic potential, and also that they can interact with other members of the gut microbiota, influencing the host’s health [7].

The possible modulating role of some members of the microbiota has been suggested, including the pathogenic bacterium *Clostridioides difficile* and the protist *Blastocystis* [7, 8]. *C*. *difficile* is an anaerobic, spore-forming bacterium, whose virulence is mediated by toxin production and some antimicrobial resistance genes [9, 10]. Some authors have pointed out that *C*. *difficile* infection (CDI) is related to a decrease in some groups of beneficial bacteria producing butyrate, which is accompanied by an increase in some groups of pathogenic bacteria belonging to the *Enterobacteriaceae* family [11]. Therefore, this bacterium is usually considered a negative modulator of the intestinal microbiota.

*Blastocystis* is an anaerobic eukaryote that exhibits high genetic diversity and is classified into 26 subtypes, whose geographical distribution and host preference are widely heterogeneous [12, 13]. In humans, the presence of ST1 to ST9 [13] and ST12 [14, 15] has been reported, while some other subtypes are commonly found in animals (such as ungulate mammals, rats and birds) [16]. Studies on the modulating role of *Blastocystis* in the microbiota have shown variable results. Some reports have linked *Blastocystis* colonization with a healthy microbiota [17], while other studies have found associations with microbiota disorders and some gastrointestinal diseases [18, 19].

Dysbiosis can be defined as changes in the communities of the microbiota that lead to a state of disease [20]. One of the consequences of CDI is a decrease in beneficial bacteria that produce butyrate, and in turn this low concentration of butyrate increase luminal oxygen concentrations, as does the abundance of pathogenic bacteria. This increase in the oxygen concentration in the intestinal lumen can prevent the colonization of *Blastocystis* because of its anaerobic nature, and therefore, finding this protist in scenarios of CDI infection is not common [7, 21]. However, *Blastocystis* may possess a mechanism to support oxidative stress in the intestine, which would allow it to continue to colonize under scenarios where its survival may otherwise be affected [22, 23]. To date, the co-occurrence of *Blastocystis* and *C*. *difficile* has been reported in a single study worldwide [24], which is interesting given that this scenario should not occur due to the alterations that CDI generates on bacterial communities and that can prevent the colonization of *Blastocystis*, as explained above.

Nonetheless, there have been no studies on gut microbiota profiling during this co-occurrence scenario. What draws our attention is this controversy in which the simultaneous colonization by *Blastocystis* and *C*. *difficile* can occur. This particularity prompted us to explore which members of the microbiota could be found in this scenario, as well as detecting changes by comparing with the members of the microbiota found in patients that only have CDI or even in patients without colonization by both microorganisms. The results obtained from this analysis would allow us to understand how *Blastocystis* may be exploiting a niche that had not been previously described and even to unveil the possible implications that this co-occurrence has on other members of the microbiota. Likewise, it would help to address questions about whether the profile of the microbiota under the co-occurrence of the mentioned microorganisms resembles that of a CDI, or if colonization by *Blastocystis* can change this profile because of the interactions that this protist may have with communities of bacteria and other eukaryotes.

Considering the lack of information about co-occurrence of *Blastocystis* and *C*. *difficile*, the present study aimed to describe the composition of the gut microbiota for three groups of patients with diarrhea and a differing *Blastocystis* and *C*. *difficile* colonization/infection status: 1. *Blastocystis* and *C*. *difficile* colonization/infection-positive patients (B+/C+), 2. *Blastocystis* colonization-negative and *C*. *difficile* infection-positive patients (B˗/C+), and 3. colonization/infection-free patients (B˗/C˗). Our results are the first to describe the bacterial and eukaryotic communities during the co-occurrence and differential presence of *Blastocystis* and *C*. *difficile* in patients with diarrhea, which helps to understand this peculiar scenario by acknowledging the microbial communities, becoming a starting point to decipher their possible interactions within the microbiota.

## Materials and methods

### Ethical statement

This study was approved by the Universidad del Rosario’s Research Ethics’ Committee (CEI-UR). This research was considered low risk due to Colombian Ministry of Health resolution 008430/1993 criteria stating that experimental interventions cannot be made regarding research subjects. Data concerning patient identification was treated confidentially, in line with Colombian legal and ethical guidelines and according to that expressed by the latest version of the Declaration of Helsinki (World Medical Association). Hospital written informed consent was obtained from patients.

### Sample selection and fecal DNA amplification for *Blastocystis* detection and subtyping

The DNA extracted from 115 fecal samples, collected by Muñoz *et al*. (2018) during a previous study that aimed characterizing *C*. *difficile* infection (CDI) in patients with diarrhea in Colombia [25], was used in the present study. The patients were selected based on symptoms of diarrhea because this is the main manifestation of CDI, but the study did not consider further clinical and sociodemographic information about the patients. DNA extraction from fecal samples was performed with the Stool DNA Isolation Kit (Norgen Biotek Corporation, Ontario, Canada), and the molecular detection of *C*. *difficile* was performed using two conventional PCR protocols targeting the 16S-rRNA and *gdh* genes, and a quantitative PCR protocol directed at 16S-rRNA. Samples were considered positive for CDI when at least one positive result was obtained with any of the aforementioned molecular tests [25]. It is important to highlight that the 115 samples were also selected based on sample volume (μL) and DNA concentration (ng/μL) optimal for the sequencing process.

For the detection of *Blastocystis* in fecal samples, the hypervariable V4 region of 18S-rRNA was amplified by conventional PCR using the primers F5 (5’-GGTCCGGTGAACACTTTGGATTT-3’) and F2 (5’- CCTACGGAAACCTTGTTACGACTTCA-3’) [26] and the following PCR cycling conditions: initial denaturation at 95° C for 10 minutes, followed by 40 cycles of 95° C for 15 seconds, 58° C for 1 minute and 72° C for 10 minutes, and a final extension at 72° C for 10 minutes. The presence of *Blastocystis* DNA was determined by observing a band of 119 base pairs (bp) following agarose gel electrophoresis.

The samples for which *Blastocystis* DNA was detected were subjected to the subtyping protocol described by Maloney *et al*. (2019) [12]. The samples were submitted to PCR for the amplification of SSU rRNA gene using one pair of modified primers that contained the Illumina overhang adapter sequences: ILMN_Blast505_532F and ILMN_Blast998_1017R. Sequencing libraries were prepared using a dual indexing strategy. A final pooled library concentration of 8 pM with 20% PhiX control was sequenced on an Illumina MiSeq using 600 cycle v3 chemistry (Illumina, San Diego, CA, USA). The resulting paired end reads were analyzed with an in-house pipeline that used the BBTools package v38.22, VSEARCH v2.8.0 [27], and BLAST+ 2.7.1. The sequences were filtered, trimmed and merged using the mentioned software. After removing singletons, clustering and the assignment of centroid sequences to Operational Taxonomic Units (OTU) was performed for each sample at 98% identity threshold. The OTUs were filtered, so those with a minimum of 100 sequences were retained and blasted against a reference database consisting of *Blastocystis* sequences. All OTUs were assigned a *Blastocystis* subtype based on the best match from the BLAST results using the consensus subtype terminology [28]. Further, the relative abundance of subtypes in each sample was calculated considering the number of reads obtained for each operational taxonomic unit (OTU), with respect to the total reads obtained per sample.

*Blastocystis* detection data were combined with *C*. *difficile* infection (CDI) data [25], and this allowed to establish three study groups: 1. colonization/infection (B+/C+, n = 31), 2. CDI only (B˗/C+, n = 44), and 3. colonization/infection-free group (B˗/C˗, n = 40). Overall, the frequency of CDI among the 115 samples was 65.22% (n = 75), whereas the frequency of *Blastocystis* was 26.96% (n = 31). The group showing colonization/infection by both organisms (B+/C+) represented 26.96% (n = 31) of the total population, the colonization/infection-free group for which the study microorganisms were not detected (B˗/C˗) represented 34.78% (n = 40) of the total population, and the B˗/C+ group represented about 40% of the total population (n = 44) (S1 Table).

### Illumina sequencing and bioinformatic analyses to determine the bacterial and eukaryotic intestinal communities

Initially, the V4 hypervariable regions of the 16S-rRNA (bacterial communities) and 18S-rRNA (eukaryotic communities) gene fragments were amplified in the 115 samples, using the 341F/806R [29] and 528F/706R [30] high resolution primers, respectively. The sizes of the fragments amplified by the aforementioned primers were 466 bp and 179 bp, respectively. The amplification described above, and the sequencing of the samples was performed by an independent entity (Novogene, Bioinformatics Technology Co., Ltd, Beijing, China). Subsequently, the construction of DNA libraries of microbial amplicons was performed using end repairing, the addition of A to tails and ligation of the index adapter. These libraries were subjected to the sequencing process on a paired-end Illumina platform (Illumina Novaseq PE250) to generate 250 bp paired-end raw reads, and assuming a minimum expected depth of 100 thousand reads per sample. Once paired-end sequences were obtained, QIIME software (version 2019.7) [31] was used to remove barcodes and primers from each pair of these demultiplexed sequences.

The taxonomic allocation of the sequences was performed using R DADA2 package (R Core Team, Vienna, Austria) [32], implementing the recommended pipeline for microbiome analysis (https://benjjneb.github.io/dada2/tutorial.html). Initially, individual reads were filtered considering a Phred score equal to or greater than 30 to minimize erroneous reads. Subsequently, forward and reverse reads were merged, and central sample inference algorithm of the reads was used to infer Amplicon Sequence Variant (ASV). Once the ASV sequence table was constructed, possible chimeras were removed from the sequences. Finally, the taxonomic allocation used the SILVA v132.16s database [33] for bacterial sequences; and the Protist Ribosomal Reference database (PR2) [34] was used to assign eukaryotic sequences. The latter contains reference sequences for protists, metazoans, embryophytes, and fungi. For the taxonomic assignments, a minimum confidence bootstrap of 50 was considered, according to the functions provided by the DADA2 package.

Additionally, when a large percentage of sequences were classified as “Unidentified”, such as the case of the 18S-rRNA sequences, a posterior identification of these sequences was conducted using BLASTn (https://blast.ncbi.nlm.nih.gov/Blast.cgi?LINK_LOC=blasthome&PAGE_TYPE=BlastSearch&PROGRAM=blastn). For this process, we generated a reference database filtering all 18S-rRNA sequences available in NCBI, specifically using the following search algorithm: 18S-rRNA [All Fields] AND (biomol_genomic[PROP] AND refseq[filter]). Also, we generated a multifasta with sequences of interest, to compare them with the constructed reference database, considering an e-value smaller than 10 and a percentage of identity of sequences greater than 95%. The resulting table contained the best assignation for the sequence (one result per sequence considering the parameters of e-value and percentage of identity), sequence accession number, the name of the organism, the e-value, and the sequence identity percentage. Once the identification was complete, this information was cross-checked with respective relative abundance obtained from DADA2 analysis.

### Descriptive and statistical analyses of the bacterial and eukaryotic communities

Descriptive analyses of the three groups of patients were performed to determine the infection and colonization/infection frequencies of the studied microorganisms in terms of percentages. Additionally, the relative frequencies of the identified subtypes of *Blastocystis* within the positive samples were determined.

First, we performed alfa diversity analyses for the ASVs corresponding to bacterial and eukaryotic communities of the three study groups. The Abundance-based Coverage Estimator (ACE) was calculated for richness estimation of the mentioned communities, and Shannon-Weaver and Simpson indexes were calculated to estimate the diversity of the communities within the three groups. Statistical differences of richness and diversity between the three groups were evaluated with a Kruskal-Wallis and a post hoc Dunn test considering Benjamini-Hochberg’s procedure (False Discovery Rate (FDR)) (p<0.05). A principal coordinate analysis (PCoA), with Bray-Curtis calculated distances, was performed for the beta diversity analyses of the ASVs corresponding to bacterial and eukaryotic communities of the three groups. Permutational analysis of variance using distance matrices (adonis) was used to evaluate statistical differences of the sample clustering depending on the study group. Alpha and beta diversity analyses were carried using R phyloseq package [35].

Further, the proportion of ASVs obtained with the DADA2 analysis was calculated considering the total number of reads per sample. Initially, we represented and described bacterial phyla with relative abundance greater than 1%, thus phyla with a lower relative abundance were categorized as “Others”. This procedure was also performed for eukaryotic classes, but instead we represented eukaryotic classes with relative abundance greater than 3%. At the family level, we represented and described bacterial families with a relative abundance greater than 10%, and eukaryotic families with a relative abundance greater than 3%. The families with a lower relative abundance were categorized as “Others”. Significant differences in the abundance of bacterial and eukaryotic families between the groups were evaluated with a Kruskal-Wallis and a post hoc Dunn test using Benjamini-Hochberg’s procedure (p<0.05).

To obtain a further description of the *Blastocystis* and *C*. *difficile* co-occurrence group (B+/C+), we created two subgroups considering the samples with colonization by a single *Blastocystis* subtype (n = 15) and with mixed subtype infections (n = 16). The description and representation of bacterial phyla of these two subgroups were performed using the phyla with a relative abundance higher than 1%, thus phyla with a lower relative abundance were categorized as “Others”. This procedure was also performed for eukaryotic classes, but instead we represented eukaryotic classes with relative abundance greater than 3%. Significant differences in the abundance of bacterial phyla and eukaryotic classes were evaluated using non-parametric Mann-Whitney test (p<0.05). On the other hand, only bacterial families of the two subgroups with a relative abundance higher than 5% were represented, and those families with a lower relative abundance were classified as “Others”. This procedure was also performed for eukaryotic families, but instead we represented eukaryotic classes with relative abundance greater than 1%. Significant differences in the abundance of these bacterial and eukaryotic families were evaluated using non-parametric Mann-Whitney test (p<0.05).

The DESeq2 package [36] was employed to assess significant differences in the abundance of bacterial and eukaryotic genera between the three study groups. A phyloseq object was used as a starting point, and then it was converted to a DESeqDataSet. After, a Wald test was applied to the dataset, and the differences in the abundance of the genera were detected by performing pairwise comparisons of the study groups. The differences were considered significant if the p-value cut-off was <0.01 (adjusted by Benjamini-Hochberg correction). Additionally, we evaluated the differences in the relative abundance of 21 bacterial genera (*Acinetobacter*, *Akkermansia*, *Alistipes*, *Bacteroides*, *Bifidobacterium*, *Bilophila*, *Dorea*, *Enterococcus*, *Escherichia/Shigella*, *Eubacterium*, *Faecalibacterium*, *Fusobacterium*, *Klebsiella*, *Methanobrevibacter*, *Parabacteroides*, *Prevotella*, *Lachnospira*, *Lachnospiraceae* groups, *Ruminococcus*, *Ruminococcaceae* groups and *Roseburia*) that could be considered as potential biomarkers of the microbiota [6, 37] using Kruskal-Wallis (p<0.05) or ANOVA test (p<0.05), according to normal distribution of the data. The *Lachnospiraceae* groups and *Ruminococcaceae* groups corresponded to undefined genera belonging to the mentioned families, and that were clustered into these categories. It is important to highlight that the selection of the potential bacterial biomarkers was performed considering what the literature has reported about the role of these bacteria, either as beneficial (e.g., butyrate producer bacteria, or bacteria with anti-inflammatory or immunomodulatory properties that help to maintain the balance of the microbiota) or as a potential pathogen.

A similar approach to the bacterial biomarkers was followed for the eukaryotic genera, where we identified all the possible of the genera of common luminal intestinal parasitic protists (CLIPPs) occurring within the groups. These protists (*Blastocystis*, *Giardia*, *Cryptosporidium*, *Dientamoeba*, *Endolimax*) were selected, since studies have reported that some CLIPPs may have pathogenic potential, and also that they can interact with other members of the gut microbiota, influencing the host’s health [7, 38]. Also, the most abundant genera of fungi and helminths were selected for this analysis. Thus, significant differences in the relative abundance of the CLIPPs between the three groups were evaluated with a Kruskal-Wallis and a post hoc Dunn test implementing Benjamini-Hochberg’s procedure (p<0.05).

Statistical differences in the relative abundance of the bacterial biomarkers, according to the *Blastocystis* subtype colonization (single infections and coexistence of different subtypes) were assessed. For this analysis, an ANOVA was performed using Tukey HSD post hoc tests (p<0.05). This same procedure was conducted with the CLIPP genera, fungi, and helminth genera. Thus, statistical differences of the mentioned eukaryotic genera, considering *Blastocystis* subtypes colonization were assessed with a Kruskal-Wallis test and a post hoc Dunn test with p-value correction using the Benjamini-Hochberg’s procedure (p<0.05).

Finally, correlation networks between the families of the most abundant bacterial phyla (Firmicutes, Bacteroidetes, and Proteobacteria) and eukaryotic divisions (Ascomycota and Basidiomycota) were performed using non-parametric Spearman correlation test. The p-values were corrected using Benjamini-Hochberg’s procedure (False Discovery Rate (FDR)), and only correlations with a correlation coefficient (ρ) above 0.6 and lower than -0.6 were considered. Afterwards, correlation networks were built using igraph, ggraph, and RCy3 R packages, and finally represented in Cytoscape 3.8.0. On the other hand, the correlation analyses were computed for the abundance of bacterial and eukaryotic genera of the most abundant families (Lachnospiraceae, Enterobacteriaceae, Ruminococcaceae, Saccharomycetales, and Exobasidiomycetales). For this analysis corr and psych packages of R were used, and the correlations between genera were performed using non-parametric Spearman correlation test, with p-values corrected with Benjamini-Hochberg’s procedure. Descriptive analyses, statistical analyses and generation of the corresponding figures were all performed using the R v.3.6.2 software package, along with Vegan v.2.5–6, phyloseq, corr, FSA, psych, igraph, ggraph, RCy3, dplyr, reshape2, tidyverse, and ggplot2 (R Core Team, Vienna, Austria).

## Results

### Study groups and relative frequencies of *Blastocystis* subtypes

**Fig 1:**
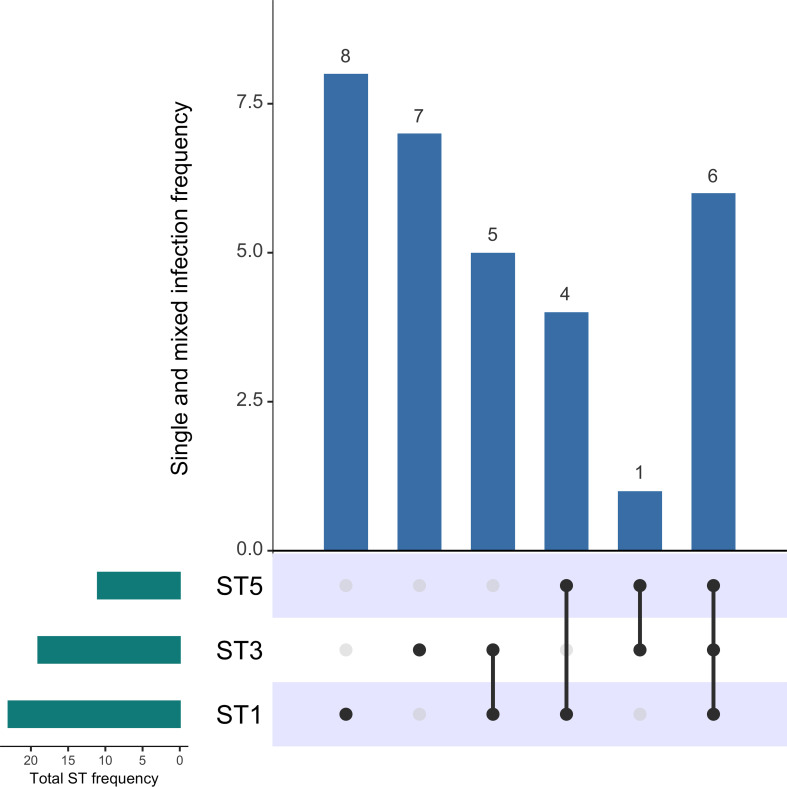
Frequency of *Blastocystis* subtypes in the 31 fecal samples (B+/C+). The absolute frequency of each ST is displayed in the lateral bar plot (green). The panel with the dots represents the single or mixed infections, which frequency is displayed in the main bar plot (blue).

For the present study, a total of 115 samples [25] belonging to patients with diarrhea (the main manifestation of CDI) were subjected to a *Blastocystis* screening. Only 31 patients out of the total presented this protist. Thus, the available data of the samples allowed the creation of the three mentioned groups: B+/C+ (26.96%, n = 31), B˗/C+ (38.26%, n = 44) and B˗/C˗ (34.78%, n = 40) (S1 Table). For the 31 samples in which *Blastocystis* was detected (B+/C+), ST1 (43.4%, n = 23) and ST3 (35.85%, n = 19) were identified as the most frequent subtypes, and ST5 (20.75%, n = 11) was identified as the third most frequent subtype. These subtypes were identified either as single infections (n = 15) or mixed infections (n = 16), where ST5 was only present in mixed infections (Fig 1 and S2 Table). For the 16 samples that presented mixed infections, up to three subtypes coexisted in the same sample (S3 Table), and the highest coexistence frequencies were for ST1/ST3/ST5 and ST1/ST3 (S2 Table).

### Description of the bacterial and eukaryotic communities of each study groups

**Fig 2:**
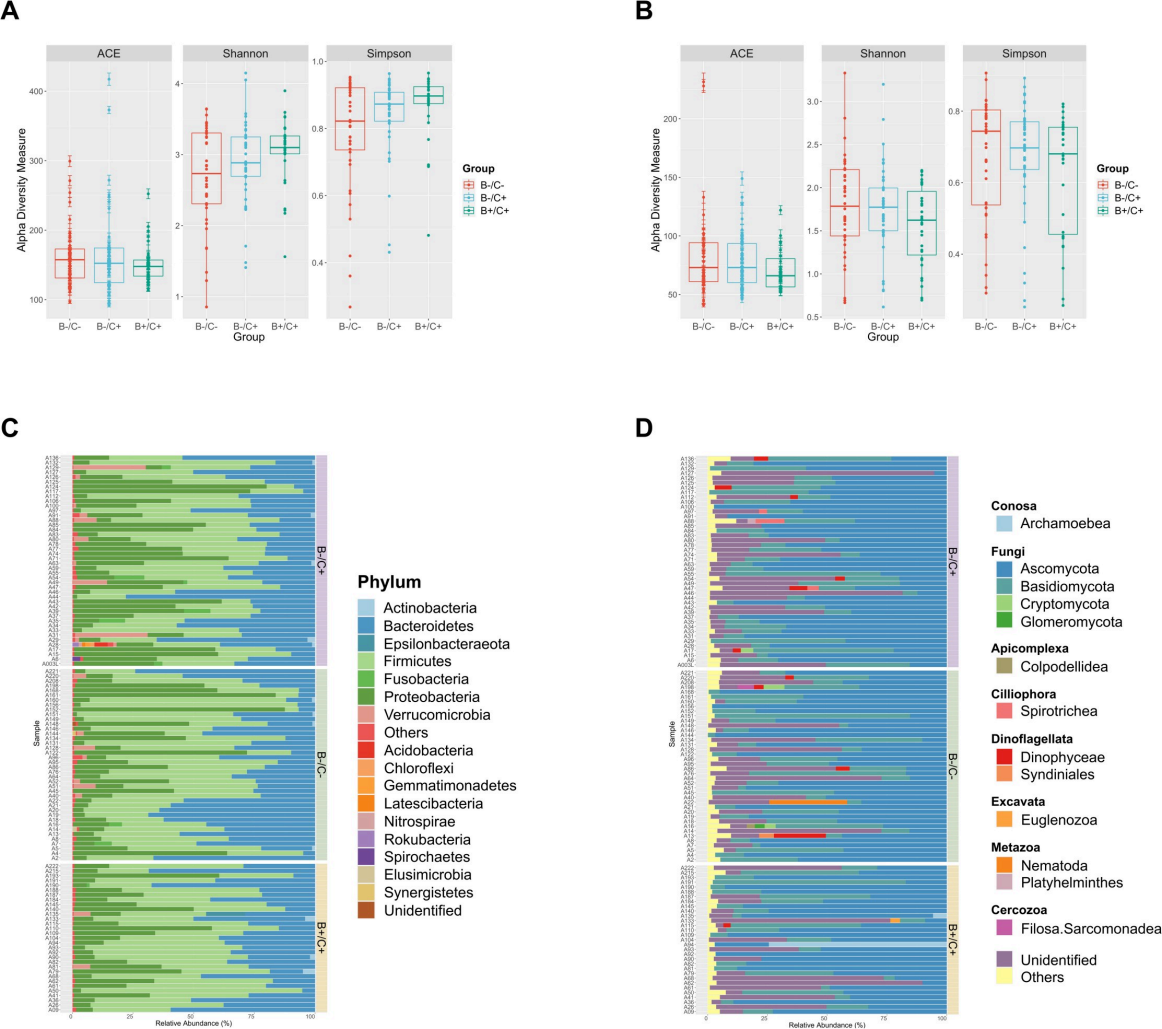
Alpha diversity measures and description of the bacterial phyla and eukaryotic divisions in the three groups. **(A)** Richness and abundance measures for the ASVs corresponding to bacterial communities of the three study groups and **(B)** Richness and abundance measures for the ASVs corresponding to eukaryotic communities of the three study groups. No significant differences were found in the richness and abundance of the bacterial and eukaryotic communities of the study groups (p<0.05). **(C)** Relative abundance of bacterial phyla identified for each of the three study groups, where the predominant phyla within each group were Firmicutes, Bacteroidetes and Proteobacteria. **(D)** Relative abundance of the eukaryotic classes identified for each of the three study groups, where the predominant classes within each group were Ascomycota and Basidiomycota.

The Abundance-based Coverage Estimator (ACE) showed a higher richness of the bacterial ASVs, compared with the richness of the eukaryotic ASVs of the three groups. The mean values obtained both for Shannon and Simpson indexes would allow to conjecture that, in general, the diversity of bacterial (Shannon index = 2.86 and Simpson index = 0.83) and eukaryotic communities (Shannon index = 1.71 and Simpson index = 0.65) was low in the three groups. The above, considering the assumption of the Simpson index in which the diversity decreases abundance, the closer the index value is to 1. Statistical analyses showed no significant differences in the richness and diversity of bacterial and eukaryotic communities of the three study groups (p>0.05, Kruskal-Wallis) (Fig 2A and 2B). On the other hand, principal coordinate analysis with Bray-Curtis calculated distances of the bacterial ASVs showed no differential clustering of the three groups (adonis, r^2^ = 0.024, p>0.05) (S1A Fig). Likewise, the principal coordinate analysis of the eukaryotic ASVs showed no differential clustering of the three groups (adonis, r^2^ = 0.008, p>0.05) (S1B Fig).

The sequencing of the 16S-rRNA V4 gene fragment of the 115 fecal samples showed that the most abundant phylum was Firmicutes and represented approximately 30% of reads for the three groups. The most abundant phyla followed by Firmicutes were Bacteroidetes and Proteobacteria, being Bacteroidetes the second most abundant in the B+/C+ and B-/C- groups, and Proteobacteria the second most abundant in the B-/C+ group (Fig 2C). The sequencing of the 18S-rRNA gene fragment allowed for the identification of different groups of fungi, protists, and other eukaryotes. For the eukaryotic community analysis, the ASVs belonging to metazoans (except helminths) and embryophytes were removed to focus the analysis on the fungal, protozoan and intestinal helminth groups because some organisms belonging to these groups are important in the hosts’ health [39]. Thus, these results showed that fungi was the most abundant phylum in the three groups and represented more than 50% of the reads, being Ascomycota (55%) and Basidiomycota (20%) the two most abundant classes of eukaryotes in the three groups (Fig 2D). The third most abundant category in the three groups represented on average 19% of the total reads and corresponded to those ASVs that were not successfully identified (“Unidentified”) both by the DADA2 taxonomic allocation process and by BLASTn further analysis. However, in BLASTn analysis 843 ASVs were identified successfully, and corresponded mainly to fungi.

At the bacterial family level, the most abundant families in the B-/C- group were: Enterobacteriaceae, Lachnospiraceae, and Bacteroideaceae, respectively (S2A Fig). Whereas, for the group B-/C+ the most abundant families were: Enterobacteriaceae, Lachnospiraceae and Ruminococcaceae, respectively. Finally, the most abundant families in the B+/C+ group were: Ruminococcaceae, Lachnospiraceae, and Bacteroideaceae, respectively. The descriptive analysis showed that only the Lachnospiraceae family was common in the three groups as one of the most abundant. No significant differences were found in the relative abundance of these families between the study groups (p>0.05, [Kruskal-Wallis]).

Despite their low abundance, we detailed the archaeal families identified in the study groups since its presence has been correlated with the host’s health status, and the abundance of some archaea may be related to diet or correlated with the presence of other members of the microbiota [40–42]. Overall, the most abundant archaeal families were *Methanobacteriaceae* (22.92%), followed by *Methanomethylophilaceae* (14.37%) and *Nitrosotaleaceae* (14.31%). Table 1 displays the mean relative abundance of the archaeal families in each one of the study groups. No significant differences were found in the relative abundance of these families between the study groups (p>0.05, [Kruskal-Wallis]), which may be due to the low relative abundance of these archaeal families in each of the groups.

**Table 1:** Mean relative abundances of archaeal families identified in the study groups.

Family	B+/C+ (Mean ± SD)	B-/C+ (Mean ± SD)	B-/C- (Mean ± SD)
Calditrichaceae	2.0e-4 ± 1.1e-3	3.9e-3 ± 2.1e-2	1.2e-3 ± 3.6e-3
Entotheonellaceae	1.0e-4 ± 5.5e-4	3.3e-4 ± 2.2e-3	3.3e-5 ± 2.1e-4
Methanobacteriaceae	0.0e0 ± 0.0e0	5.9e-3 ± 1.9e-2	7.1e-3 ± 3.3e-2
Methanocellaceae	0.0e0 ± 0.0e0	5.0e-4 ± 3.3e-3	0.0e0 ± 0.0e0
Methanofastidiosaceae	0.0e0 ± 0.0e0	1.2e-3 ± 7.6e-3	6.3e-5 ± 3.9e-4
Methanomassiliicoccaceae	0.0e0 ± 0.0e0	0.0e0 ± 0.0e0	2.0e-4 ± 1.3e-3
Methanomethylophilaceae	1.6e-3 ± 8.6e-3	2.0e-3 ± 7.9e-3	5.1e-3 ± 2.5e-2
Methanoregulaceae	0.0e0 ± 0.0e0	2.5e-3 ± 1.6e-2	1.1e-4 ± 6.9e-4
Methanosaetaceae	0.0e0 ± 0.0e0	5.2e-3 ± 3.1e-2	1.1e-3 ± 5.7e-3
Methanosarcinaceae	0.0e0 ± 0.0e0	3.8e-4 ± 2.5e-3	0.0e0 ± 0.0e0
Methanospirillaceae	0.0e0 ± 0.0e0	1.0e-3 ± 6.7e-3	9.4e-5 ± 5.9e-4
Nitrosopumilaceae	0.0e0 ± 0.0e0	6.5e-3 ± 4.3e-2	4.3e-4 ± 1.9e-3
Nitrososphaeraceae	0.0e0 ± 0.0e0	1.3e-3 ± 8.7e-3	0.0e0 ± 0.0e0
Nitrosotaleaceae	0.0e0 ± 0.0e0	7.6e-3 ± 5.0e-2	3.7e-5 ± 2.3e-4

Regarding eukaryotic families, the descriptive analysis showed that the most abundant eukaryotic family in the three groups was Saccharomycetales, which accounted for more than 50% of the reads on average. Additionally, the third most abundant family in the three groups, followed by the "Unidentified" family category corresponded to Exobasidiomycetales, which represented on average 15% of the reads (S2B Fig). However, no significant differences were found in the relative abundance of these families between the study groups (p>0.05, [Kruskal-Wallis]).

A description of the bacterial and eukaryotic communities within the B+/C+ group was also performed, considering two subgroups: samples with colonization by a single *Blastocystis* subtype and mixed subtype infections. The relative abundance of the bacterial phyla and the eukaryotic classes found in these two subgroups are displayed in the S3A and S3B Fig.

At the family level, the most abundant bacterial families within the samples with mixed subtype infections were Bacteroidaceae, Ruminococcaceae, and Lachnospiraceae, respectively. Whereas, within the samples with colonization by a single subtype, the most abundant families were Ruminococcaceae, Lachnospiraceae, and Prevotellaceae, respectively (S3C Fig). Statistical analyzes showed significant differences abundance of the family Prevotellaceae, where it displays a higher abundance within samples with colonization by a single subtype compared to the samples with mixed subtype infections (p = 0.031, [Mann-Whitney]) (S4 Fig). Additionally, statistical differences were found for the Pseudomonadaceae family, where this family showed a higher relative abundance within the samples with mixed subtype infection compared to those with colonization by a single subtype (p = 0.033, [Mann-Whitney]) (S4 Fig). Regarding eukaryotic families, it was observed that Saccharomycetales and Exobasidiomycetales were the most abundant families in both of the subgroups, respectively (S3D Fig). However, no significant differences were observed in the relative abundances of these families between the subgroups (p>0.05, [Mann-Whitney]).

### Analysis of bacterial biomarkers, fungi, nematodes and Common Luminal Intestinal Parasitic Protists (CLIPPs) of the study groups

Initially, the DESeq analysis showed that the abundance of 5 bacterial genera and two bacterial ASVs unclassified at the genus level were significantly diminished in the B+/C+ group compared to the B-/C+ and B-/C- groups (DESeq2, Benjamini-Hochberg correction p-value <0.01). These differences were due to the low abundance of these genera and ASVs in the B+/C+ group, principally when compared with the B-/C- group, where most of the differences were found (S4 Table). Additionally, the comparison between the groups B-/C+ and B-/C- did not show any significant difference in the abundance of bacterial genera, and therefore it was not displayed in the S4 Table (DESeq2, Benjamini-Hochberg correction p-value >0.01).

**Fig 3:**
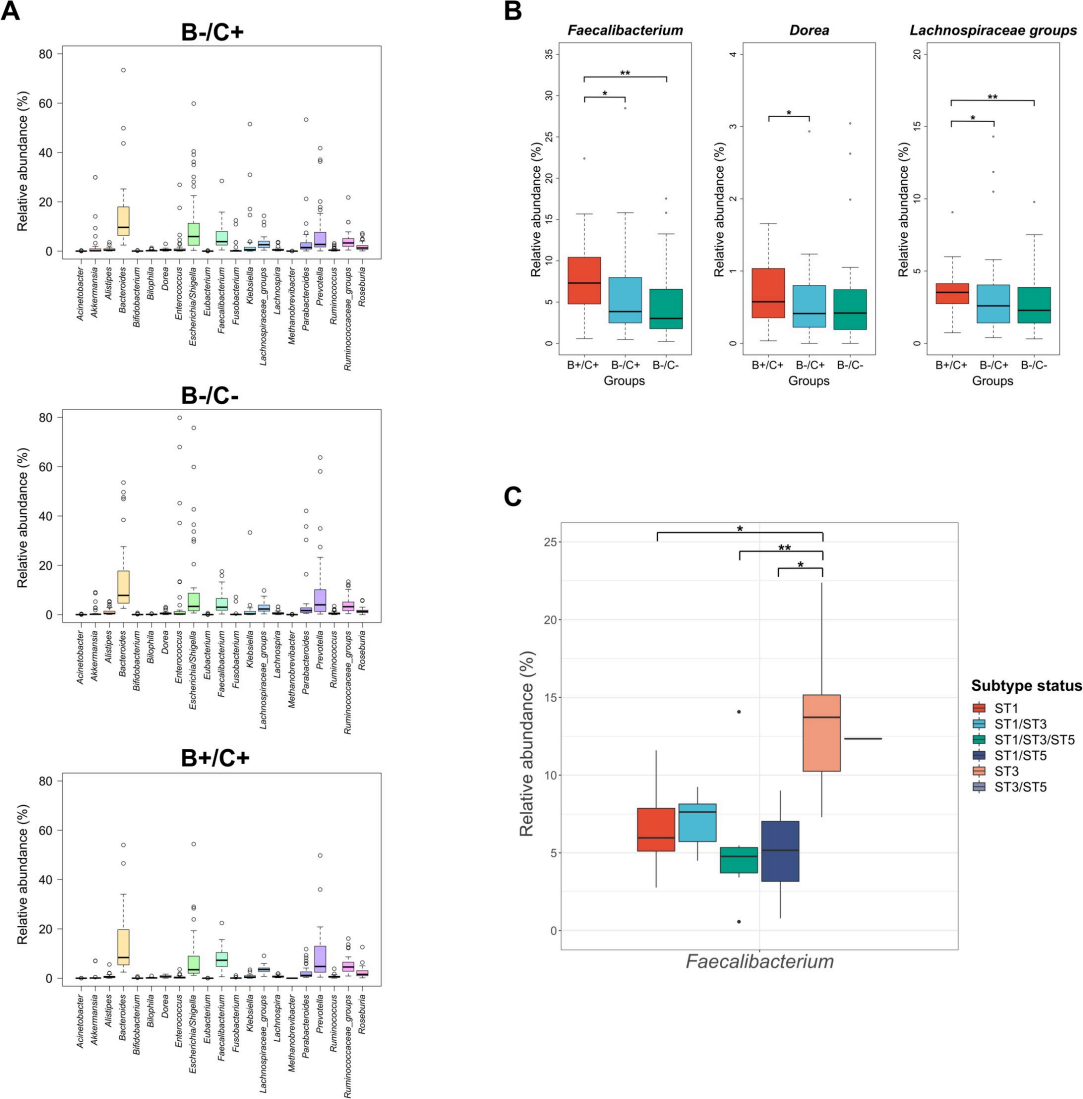
Description and evaluation of the statistical differences in the abundance of bacterial biomarkers among the three study groups. **(A)** Description of the relative abundance of 21 genera of bacteria considered as possible biomarkers of the intestinal microbiota among the three study groups. **(B)** Three genera of bacterial biomarkers presented significant differences in their relative abundance among some of the study groups, where all of these genera display a higher abundance in the B+/C+ group. **(C)** Changes in the relative abundance of *Faecalibacterium* considering the colonization of a determined *Blastocystis* subtype, showing that this genus has a higher abundance in ST3 colonized patients. Significant differences between the study groups were evaluated using Kruskal-Wallis test and ANOVA (*, p<0.05; **, p<0.01).

For further analyses of the possible changes in the bacterial communities of the three study groups, we evaluated the differences in the relative abundances of specific genera of bacteria, and of some genera of eukaryotes that can play important roles within the microbiota. Regarding bacterial communities, 21 bacterial genera acknowledged as potential biomarkers of the microbiota were selected [6, 37] (Fig 3A). The statistical analyses showed significant differences only in the relative abundance of *Faecalibacterium*, *Dorea*, and in groups of undefined genera belonging to the family Lachnospiraceae (*Lachnospiraceae* groups) among the three study groups. Thus, the mentioned genera exhibited a higher relative abundance within the B+/C+ group compared to the B-/C+ group (p<0.05, [Kruskal-Wallis]). Also, the relative abundance of the *Faecalibacterium* genus and *Lachnospiraceae* groups was significantly higher within the B+/C+ group compared to B-/C- group (p<0.01, [Kruskal-Wallis]) (Fig 3B).

In the case of the eukaryotic communities of the three groups, the DESeq analysis showed that three fungi genera were significantly diminished in the B+/C+ group compared to the B-/C- group (DESeq2, Benajmini-Hochberg correction p-value <0.01) (S4 Table). The only significant difference found between the B-/C+ and B-/C- groups corresponded to the diminished abundance of the genus *Coniochaeta* in the B-/C+ group (fold change = -26.51, p-value = 1.03e-21). On the other hand, only three genera considered as CLIPPs were identified, namely *Entamoeba*, *Endolimax*, and *Cryptosporidium* [38]. Additionally, the three most abundant genera of fungi (*Candida*, *Saccharomyces*, and *Malassezia*) and the most abundant genus of nematodes (*Strongyloides*) were selected. Table 2 shows the value of the mean abundance of the seven genera mentioned above, together with their respective standard deviations (SD). The statistical analyses showed no significant differences in the abundance of these genera between the study groups (p>0.05, [Kruskal-Wallis]).

**Table 2:** Mean relative abundance of the seven genera of eukaryotes (CLIPPs, nematodes and fungi) selected for the three study groups.

Genus	B+/C+ (Mean ± DS)	B˗/C+ (Mean ± DS)	B˗/C˗ (Mean ± DS)
*Candida*	27.62 ± 24.83	24.48 ± 22.73	21.41 ± 20.31
*Malassezia*	15.67 ± 16.56	16.92 ± 14.49	17.64 ± 17.20
*Saccharomyces*	21.04 ± 17.33	21.06 ± 18.23	23.26 ± 20.51
*Entamoeba*	2.65 ± 13.14	4.05e-2 ± 0.11	8.88e-2 ± 0.36
*Endolimax*	1.53e-3 ± 8.35e-3	3.53e-4 ± 2.31e-3	7.0e-5 ± 4.37e-4
*Cryptosporidium*	1.25e-2 ± 3.02e-2	4.10e-3 ± 2.01e-2	1.31e-2 ± 4.03e-2
*Strongyloides*	2.43e-2 ± 0.12	2.0e-2 ± 0.13	0.85 ± 5.08

Additionally, within the samples of the B+/C+ group, the possible changes in the abundance of the 21 bacterial biomarkers and the seven selected eukaryotic genera were evaluated, considering single infections and coexistence of *Blastocystis* subtypes (S5 and S6 Figs). In the case of bacterial biomarkers, the statistical analyses showed only significant differences in the relative abundance of *Faecalibacterium*, considering *Blastocystis* subtypes. The relative abundance of this genus was significantly higher in the samples with colonization by ST3 than in the samples with ST1 (p = 0.017, [ANOVA]), ST1/ST3/ST5 coexistence (p = 0.011, [ANOVA]), and ST1/ST5 coexistence (p = 0.018, [ANOVA]) (Fig 3C). On the other hand, the statistical analyses showed no significant differences in the relative abundances of the seven eukaryotic genera, considering single infections or coexistence of *Blastocystis* subtypes [p>0.05, [Kruskal-Wallis]).

### Correlations between bacterial and eukaryotic communities are different among study groups

**Fig 4:**
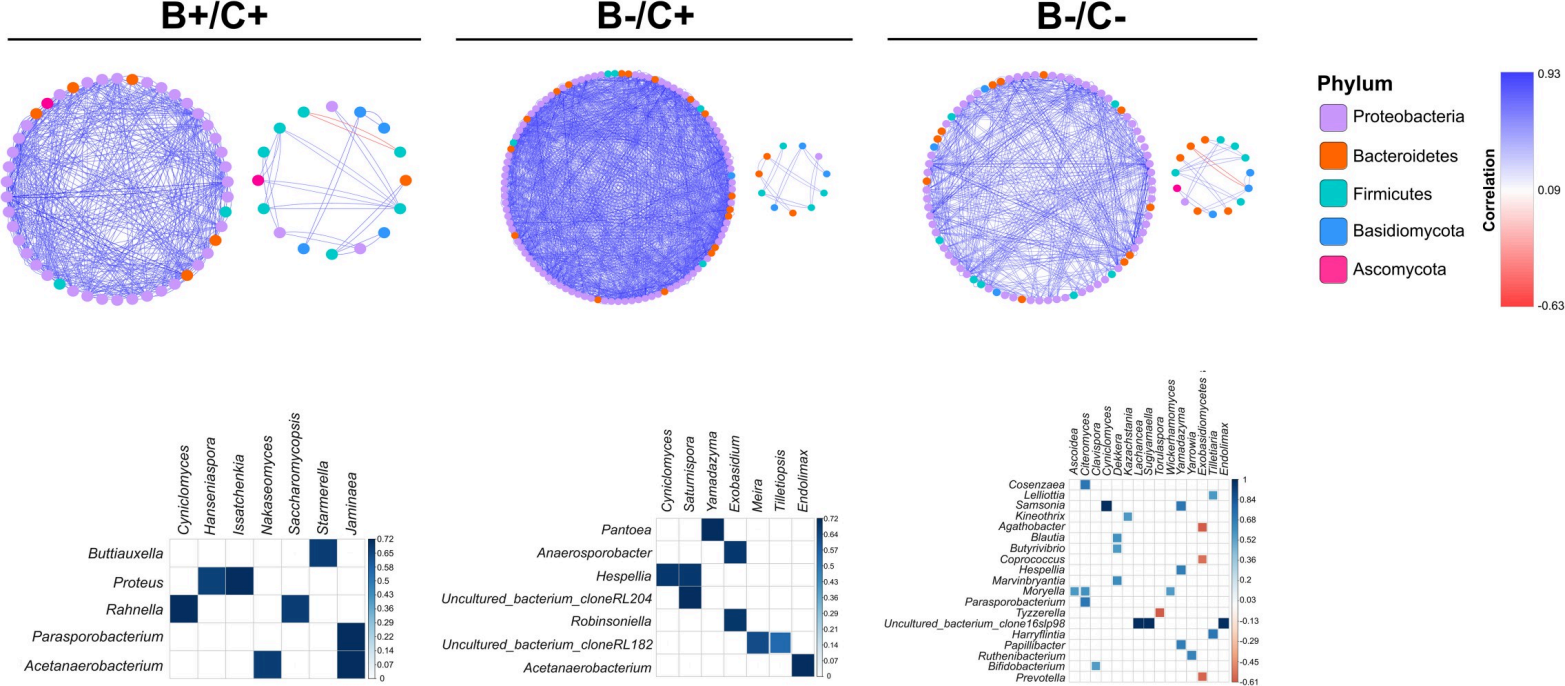
Intra-domain and inter-domain correlations in the three study groups. Correlation networks of the B+/C+ (top left), B-/C+ (top center), and B-/C- (top right) groups. The nodes represent the bacterial and eukaryotic families, and its color represents the phylum to which it belongs. Blue edges and red edges indicate positive and negative correlations, respectively. The networks only represent significant correlations (p<0.05, after FDR correction) with ρ above 0.6 and lower than -0.6, and that were computed with the Spearman correlation test. This figure also displays correlation plots of the genera belonging to the most abundant bacterial and eukaryotic families of the B+/C+ (bottom left), B-/C+ (bottom center), and B-/C- (bottom right) groups. Blue and red squares represent positive and negative correlations, respectively. Only significant correlations, computed with the Spearman correlation test, (p<0.05, after FDR correction) were represented in the mentioned plots.

To evaluate possible interactions between bacterial and eukaryotic communities of the three study groups, we performed a correlation network of the families belonging to the most abundant bacterial phyla (Firmicutes, Bacteroidetes and Proteobacteria) and the most abundant eukaryotic divisions (Ascomycota and Basidiomycota). The correlation network was performed based on the Spearman correlation test, where Fig 4 shows only significant correlations (p<0.05 after FDR correction) with a correlation coefficient (ρ) lower than -0.6 and higher than 0.6 (Fig 4, top). Despite that, most of the correlations were positive and mainly between families of the Proteobacteria phylum, the networks of the groups were notably different from each other.

Only B+/C+ and B-/C- groups displayed moderate negative correlations between some of the bacterial and eukaryotic families (Fig 4, top left and top right). The network of the B-/C+ group was markedly different from the networks of the other two groups since this network had the highest number of strong correlations, mainly between the families of Proteobacteria and Bacteroidetes phyla (Fig 4, top center). Additionally, it is interesting to observe a slight clustering in the networks of each group, where different interactions from the ones presented in the main network were observed. For instance, instead of representing interactions mostly between families of Proteobacteria, these clusters exhibited interactions between Firmicutes, Bacteroidetes, and Basidiomycota.

In the B+/C+ group was notable that the families of the Basidiomycota division correlated mostly with each other, while families of Ascomycota correlated with Proteobacteria and Firmicutes (Fig 4, top left). Conversely, in the B-/C- group, Basidiomycota families correlated with each other as well as with families of Proteobacteria phylum (Fig 4, top right). In general, it can be observed that there are not many correlations between bacterial families and families of the Ascomycota division; in fact, in the group B-/C+ no correlation involving this division of fungi was evidenced (Fig 4, top center).

On the other hand, we computed correlations on the genera belonging to the most abundant families of bacteria (Lachnospiraceae, Ruminococcaceae, and Enterobacteriaceae) and eukaryotes (Saccharomycetales and Exobasidiomycetales) of the three study groups. Also, the 21 bacterial genera considered as biomarkers, and the seven genera of eukaryotes selected for previous analyses were added to the correlation analysis (Fig 4, bottom). The results showed a higher number of statistically significant correlations (ρ>0.5, p<0.05 after FDR correction) between genera of bacteria and eukaryotes in the B-/C- group (number of correlations: 24) (Fig 4, bottom right), compared to B-/C+ (number of correlations: 9) (Fig 4, bottom center) and B+/C+ groups (number of correlations: 8) (Fig 4, bottom left).

Within the B+/C+ group, most of the significant correlations were displayed between fungi genera belonging to the Saccharomycetales family and bacteria belonging to the Enterobacteriaceae family (Fig 4, bottom left). The B-/C+ group presented mostly correlations between the fungi genera of the two evaluated families and genera of the Lachnospiraceae family (Fig 4, bottom center). Finally, in the B-/C- group, it was observed that various genera of the three bacterial families were correlated mainly with genera of the Saccharomycetales family. Also, this group was the only that displayed moderate inverse correlations (ρ = -0.6, p<0.01 after FDR correction); for instance, these correlations were observed between *Exobasidiomycetes* and Lachnospiraceae genera (*Agathobacter* and *Coprococcus*), as well as between *Exobasidiomyctes* and *Prevotella* (Fig 4, bottom right).

## Discussion

It is currently known that the pathogenic bacterium *C*. *difficile* usually causes diarrhea associated with the use of antibiotics, which is related to a decrease in the abundance of beneficial bacteria in the intestinal microbiota [11]. However, *Blastocystis* is a protist whose role as a pathogen or commensal within the intestinal microbiota has not been defined, and therefore it is not completely known how it can modulate and interact with the communities of bacteria and other intestinal eukaryotes [17–19]. The coexistence of *Blastocystis* and *C*. *difficile* has only been reported once [24], and therefore it is interesting to know the bacterial and eukaryotic communities that can be found under this scenario, given that colonization by *Blastocystis* is not usually reported in scenarios of decrease of beneficial bacteria as under CDI [7]. Additionally, few studies have focused on the description of the intestinal eukaryotic communities in certain states of the host’s health. Therefore, this study aimed to describe for the first time the communities of intestinal bacteria and eukaryotes within three specific groups of patients (B+/C+, B-/C+ and B-/C-) who presented diarrhea.

First, it is important to highlight the limitations of the present study. Metadata about the patients that would have allowed for other factors that influence the composition of the microbiota (e.g., diet, age, sex, body mass index, prior medical treatments) was not available. Because all of the patients belonging to the three groups presented with diarrhea, there was no group without any physiological imbalance in the microbiota that would allow for broader comparisons. Furthermore, the data of the present study did not allow the creation of a group with colonization by *Blastocystis* only (B+/C˗), that would also have allowed for more robust comparisons of the bacterial and eukaryotic communities between groups. Future studies are therefore needed to confirm our preliminary findings from microbiota profiling.

Herein we show a colonization/infection frequency of 26.96% by *Blastocystis* and *C*. *difficile*. The results obtained from the subtyping of *Blastocystis* were consistent with those reported in other studies, where it is described that ST1 and ST3 are some of the most abundant subtypes in the human intestine worldwide, which includes Colombia as shown in our study [14, 43]. A striking finding in our study was the detection of ST5, which was the first report of this subtype in Colombia and, interestingly was found only in mixed subtype infections (Fig 1). The finding of mixed subtype infections supports the advantage of next generation sequencing in the subtyping of *Blastocystis* because this technique offers high resolution and sensitivity [12]. Therefore, it is likely that ST5 had not been previously reported in Colombia because studies in the country had only employed Sanger sequencing for subtyping [14, 43–45]; a method that has less sensitivity in the detection of multiple *Blastocystis* subtypes compared to next generation sequencing [12].

Although there were no significant differences in the richness and diversity of the ASVs corresponding to the bacterial communities of the three study groups, the Simpson index and ACE could suggest an overall low bacterial diversity in the three groups (Fig 2A and S1A Fig). This potential low bacterial diversity could be related to the effects of CDI in the microbiota, where there is a decrease in bacterial abundance, especially of beneficial ones [46]. As in the bacterial communities, the analyzes of alpha and beta diversity of the ASVs corresponding to the eukaryotic communities did not show significant differences between the study groups (Fig 2B and S1B Fig). Nonetheless, ACE and Simpson index may suggest an overall low eukaryotic diversity in the three groups. Few studies have addressed the changes of eukaryotic communities in CDI infection, but it has been suggested that imbalances in the microbiota can significantly alter fungal communities causing fungal dysbiosis [47, 48]. As a perspective, this should be explored in the patients presented here.

On the other hand, our results showed that the relative abundance of archaea families is low within the three study groups. The low abundance of methanogenic archaea (Table 1) indicates that the microbiota of the patients belonging to the three groups may display a functional imbalance. The uptake of hydrogen from some methanogenic archaea can drive the processes of primary and secondary fermenters within the microbiota. Moreover, a direct correlation between methane production and constipation has been reported, which also supports the fact that the patients belonging to the three study groups presented diarrhea [41, 49, 50]. For the B+/C+ group, a higher relative abundance of archaea was expected because some studies have reported significant associations between *Blastocystis* colonization and the abundance of some archaea [51, 52]. Because of the above, it could be hypothesized that the low relative abundance of archaea in the three study groups may be mainly driven by the low abundance of bacteria (Fig 2A). This, considering the syntrophic relationship that has been described between these organisms, because archaea can optimize the metabolic pathways of fermenting bacteria [53]. Future studies are required to assess this hypothesis and observe how the syntrophic relation between bacteria and archaea could be changing in this scenario. However, fermentation may not be completely restricted due to the presence of butyrate-producing bacteria in the three study groups, although it could be occurring at a very low rate. Therefore, archaea could be competing in an environment with limited resources (H_2_). Still, future studies are needed to elucidate the ecological relationships between archaea, whether if they are competing or coexisting in a limited resource environment [54]. Also, as a perspective, it would be interesting to acknowledge if a possible predominance of a determined methanogenesis type (hydrogenotrophic, methylotrophic, and acetotrophic) [40] is occurring in the scenario of the three groups.

Regarding eukaryotic communities of the study groups, descriptive analyses showed that the three groups presented a high abundance of fungi, especially of genera belonging to the Saccharomycetales family. To date, there is no clear information about how *Blastocystis* can interact or modify fungal communities of the microbiota when an intestinal imbalance is present. One of the few studies showed that colonization by *Blastocystis* increased yeast abundance [55]. However, in the case of our study groups, the lack of statistically significant results makes it difficult to establish possible interactions between *Blastocystis* and some fungi genera.

The description of the bacterial phyla within the subgroups with colonization by a subtype and with mixed infection was very similar to the three study groups (B+/C+, B-/C+ and B-/C-), where the Firmicutes, Bacteroidetes, and Proteobacteria phyla were predominant (S3A Fig). The same case was observed for the eukaryotic classes, where Ascomycota and Basidiomycota were the most abundant classes in the two subgroups (S3B Fig). It is interesting to observe that within this scenario of *Blastocystis* and *C*. *difficile* co-occurrence, the samples with mixed subtype infections show a greater abundance of a family of the phylum Proteobacteria (S4 Fig), specifically Pseudomonadaceae. Whereas when colonization by a single subtype of *Blastocystis* occurs, there is a greater abundance of a potentially beneficial family, namely Prevotellaceae (S4 Fig).

The DESeq analysis applied to the bacterial genera showed a diminished abundance of *Akkermansia* in the B+/C+ group (S4 Table), which is in accordance with that described by other studies where the presence of *Blastocystis* was related to a low abundance of *Akkermansia* [52, 56]. Also, significant differences were observed in the abundances of beneficial bacteria genera (*Faecalibacterium*, *Dorea* and groups of undefined genera belonging to the family Lachnospiraceae) between the three study groups (Fig 3B). Some studies have suggested that colonization by a determined *Blastocystis* subtype can be related with the decrease or increase of some bacterial genera [52, 57]. The study by Gabrielli et al., (2020) reported a higher abundance of some genera of beneficial bacteria such as *Prevotella* and *Ruminococcus* in patients colonized by *Blastocystis* ST3 [58]. In the case of the results of our study, it is interesting to observe that the abundance of the *Faecalibacterium* genus was significantly higher in the samples of patients who presented colonization by ST3 (Fig 3C). The significantly higher abundance of these three genera within the B+/C+ group could again suggest a predominance of beneficial bacterial genera when compared to the other two groups.

It could even be hypothesized that the presence of *Blastocystis* in cases of CDI can cause a change in some groups of bacteria, increasing the abundance of beneficial bacteria (e.g., butyrate producers). The high abundance of these genera (*Faecalibacterium*, *Dorea*, and *Lachnospiraceae* groups) in the B+/C+ group can be explained by the hypothesis of the predatory role of *Blastocystis*, where an increase in the richness of some beneficial bacterial groups is the result of *Blastocystis* predation over some other groups of bacteria [59].

The analyses of the relative abundance of the genera of fungi, nematodes and CLIPPs did not show to be significant between the study groups (Table 2). However, the DESeq analysis showed a significantly low abundance of the genera *Antrodia* and *Clavispora* in the B+/C+ group compared to the other two study groups (S4 Table). The information about the possible role of these fungi in the gut microbiota is limited. Still, *Antrodia* has been described as a genus that harbors some species with potential anti-inflammatory, hepato-protective and immunomodulatory properties [60, 61]. Regarding *Clavispora*, it is possible to hypothesize that this genus may be linked to some disease states since it has been found enriched in samples of patients with Crohn’s disease [62].

The detection of only a few CLIPPs in the samples may be explained by the fact that microorganisms such as *Giardia* and *Dientamoeba*, do not possess hydrogenosomes that allow them to survive under the aerobic environment of a disturbed microbiota [63, 64]. Future studies should evaluate the effect of these genera of parasites (*Giardia* and *Dientamoeba*) on the bacterial communities of our three groups to obtain a broader understanding of the interactions between these taxonomic units within the microbiome. Because most microbiota studies focus on the bacterial communities, information on the effect of a disturbed gut because of CDI [65], or the presence of *Blastocystis* on fungal and other eukaryotic communities is limited [66]. Thus, our results tried to contribute toward an understanding of the eukaryotic communities, specifically within a specific group of patients that harbor a physiological imbalance in their microbiota.

Overall, the correlation networks showed a predominance of the interaction between Proteobacteria families, as well as a low interaction between bacterial families and fungal families (Fig 4, top). Additionally, a slight clustering in the three correlation networks could suggest a shortage of cooperation in the whole system [67], considering the lack of connection between families of the cluster with the main network, and the low or absence of interaction with fungal families. However, future studies are needed to elucidate the complex polymicrobial interactions of the microbiota [68] in these specific scenarios. Also, it would be interesting to address the possible implications of these interactions upon the pre-existing condition (intestinal imbalance) of the three types of patients.

Most of the correlations between the abundance of specific bacterial and eukaryotic genera showed to be positive correlations (Fig 4, bottom); this could suggest that an antagonistic interaction between these organisms may not be present. Nonetheless, establishing definite roles for the fungi communities of the microbiota in a scenario of intestinal imbalance is difficult due to the limitations in the information of fungal communities and their interactions in the microbiota. Few studies have suggested that some of these yeasts could have a probiotic effect, and even some yeast species have been studied in the prevention of many bacterial pathogen colonization, among them *C*. *difficile* [69–71]. Conversely, studies have suggested that commensal fungi can undergo a shift towards pathogen organisms, that can even exacerbate a pre-existing disease [72]. This shift could be influenced by multiple predisposing factors such as chemical, nutritional or physiological alterations [73]. Future approaches implementing models can help to unravel the possible role of these fungi communities within the microbiota of the three study groups, observing whether if they have a beneficial or pathogenic role.

Despite the limitations of the present study, herein we report the first description of bacterial and eukaryotic communities of three specific groups of patients exhibiting diarrhea, highlighting the scenario of *Blastocystis* and *C*. *difficile* co-occurrence. In addition, our results may suggest that the presence of *Blastocystis* in patients with CDI (B+/C+) can generate a possible shift towards more beneficial bacterial groups. Therefore, the results of our study may support the hypothesis that *Blastocystis*, as a commensal prototype of the microbiome, may favor the proliferation of potentially beneficial bacteria, such as butyrate producers or bacteria with anti-inflammatory properties (i.e., *Faecalibacterium*, *Dorea* and *Lachnospiraceae* groups). Finally, our results hope to encourage future microbiota studies to analyze eukaryotic communities thoroughly, since their interaction with bacteria and other eukaryotes would give more information about the functioning of this complex environment under specific scenarios.

## Supporting information

S1 Table Frequencies of the three study groups that comprise the overall population (n = 115). (PDF)

S2 Table Relative frequency of *Blastocystis* subtypes in single infections and mixed subtype infections within the 31 *Blastocystis* positive samples. (PDF)

S3 Table Relative abundance of *Blastocystis* subtypes within each positive sample. (PDF)

S4 Table Bacterial and eukaryotic genera found to be significantly different by DESeq analysis between the three study groups. (PDF)

S1 Fig Beta diversity of the bacterial and eukaryotic ASVs of the three study groups. **(A)** Principal coordinate analysis (PCoA) of the bacterial ASVs. **(B)** Principal coordinate analysis (PCoA) of the eukaryotic ASVs of the three study groups. The percentage of variation explained by the two dimensions of the PCoA is displayed on the axis.
(TIF)

S2 Fig Heatmap of the relative abundance of the most abundant bacterial and eukaryotic families within the study groups. **(A)** Relative abundance of the 24 most abundant bacterial families of the three study groups. **(B)** Relative abundance of the 21 most abundant eukaryotic families of the three study groups.
(TIF)

S3 Fig Description of some bacterial and eukaryotic taxonomic groups present in the samples with mixed subtype infections and with colonization by a single subtype. **(A)** Bar plot of the relative abundance of the bacterial phyla identified in the two subgroups, where Firmicutes, Bacteroidetes, and Proteobacteria are the most abundant. **(B)** Bar plot of the relative abundance of the eukaryotic classes identified in the two subgroups, where Ascomycota and Basidiomycota where the most abundant. **(C)** Heatmap of the relative abundance of the 30 most abundant bacterial families identified in the two subgroups. **(D)** Heatmap of the relative abundance of the 15 most abundant eukaryotic families identified in the two subgroups.
(TIF)

S4 Fig Differential relative abundance of Prevotellaceae and Pseudomonadaceae families between the mixed subtype subgroup and the subgroup with colonization by a single *Blastocystis* subtype. Significant differences between the study groups were evaluated using non-parametric Mann-Whitney test (*, p < 0.05).
(TIF)

S5 Fig Relative abundance of the bacterial biomarkers considering colonization by a single subtype of *Blastocystis* or by coexistence of different subtypes (subtype status). For the boxplot the genera: *Acinetobacter*, *Akkermansia*, *Bifidobacterium*, *Bilophila*, *Eubacterium*, *Fusobacterium* and *Methanobrevibacter* were not displayed since their relative abundance was low.
(TIF)

S6 Fig Relative abundance of the seven eukaryotic genera (CLIPPs, fungi genera, and nematode genus) considering colonization by a single subtype of *Blastocystis* or by coexistence of different subtypes (subtype status). (TIF)
