# Supplementary material for: Gut microbiota profiles in diarrheic patients with co-occurrence of Clostridioides difficile and Blastocystis
Source: PLoS One. 2021 Mar 16;16(3):e0248185. doi: 10.1371/journal.pone.0248185 (PMC7963057; doi:10.1371/journal.pone.0248185)
Supplement: S1 Table — (PDF) [file pone.0248185.s001.pdf]

S1 Table.

Group	Sample size (n=115)	Frequency
B+/C+	31	26.96
B-/C+	44	38.26
B-/C-	40	34.78
