# Supplementary material for: Gut microbiota profiles in diarrheic patients with co-occurrence of Clostridioides difficile and Blastocystis
Source: PLoS One. 2021 Mar 16;16(3):e0248185. doi: 10.1371/journal.pone.0248185 (PMC7963057; doi:10.1371/journal.pone.0248185)
Supplement: S2 Table — (PDF) [file pone.0248185.s002.pdf]

S2 Table.

	Single subtype infections (n=15)			Mixed subtype infections (n=16)			
	**ST1**	**ST3**	**ST5**	**ST1/ST3**	**ST1/ST5**	**ST5/ST3**	**ST1/ST3/ST5**
**Relative frequency (%)**	53.33	46.67	0.0	31.25	25.0	6.25	37.5
