# Supplementary material for: Gut microbiota profiles in diarrheic patients with co-occurrence of Clostridioides difficile and Blastocystis
Source: PLoS One. 2021 Mar 16;16(3):e0248185. doi: 10.1371/journal.pone.0248185 (PMC7963057; doi:10.1371/journal.pone.0248185)
Supplement: S3 Table — (PDF) [file pone.0248185.s003.pdf]

S3 Table.

Sample	ST1	ST3	ST5
A09	0.3	99.7	0
A26	0	99.62	0.38
A36	100	0	0
A41	0	100	0
A50	98.73	0	1.27
A61	100	0	0
A62	19.43	36.41	44.16
A68	99.89	0.11	0
A79	46.43	53.57	0
A81	70.13	0	29.87
A82	87.25	0	12.75
A90	0	100	0
A92	0	100	0
A93	0	100	0
A94	2.29	52.63	45.08
A104	99.03	0.38	0.58
A109	100	0	0
A110	100	0	0
A115	100	0	0
A133	100	0	0
A135	100	0	0
A140	0.15	98.55	1.3
A145	97.81	0	2.19
A184	100	0	0
A187	0	100	0
A188	99.52	0.48	0
A190	60.47	22.22	17.31
A191	0	100	0
A193	0.7	99.3	0
A215	97.2	2.58	0.22
A222	0	100	0
