# Supplementary material for: Gut microbiota profiles in diarrheic patients with co-occurrence of Clostridioides difficile and Blastocystis
Source: PLoS One. 2021 Mar 16;16(3):e0248185. doi: 10.1371/journal.pone.0248185 (PMC7963057; doi:10.1371/journal.pone.0248185)
Supplement: S4 Table — (PDF) [file pone.0248185.s004.pdf]

S4 Table.

Kingdom	Genus	B+/C+ vs. B-/C+		B+/C+ vs. B-/C-	
		Log2 (fold change)	p-value	Log2 (fold change)	p-value
Bacteria	*Cetobacterium*	-23.74	2.92e-17	-23.53	2.99e-16
	Tissierella	-15.36	1.52e-04	-24.44	3.67e-12
	Akkermansia	-2.83	4.59e-04	-	-
	Enterococcus	-	-	-5.36	1.35e-11
	Escherichia/Shigella	-	-	-1.81	4.33e-03
	Clostridiales_vadinBB60_group[a]	-	-	-1.69	3.15e-03
	Unidentified bacterium[b]	-	-	-2.19	9.37e-04
Eukaryota	*Antrodia*	-22.28	4.82e-18	-21.63	3.16e-16
	Coniochaeta	-	-	-22.7	2.65e-11
	Clavispora	-	-	-3.73	3.56e-03

[a] ASV unclassified at the genus level but belonging to the family mentioned above.
[b] ASV unclassified at phylum level.
