# Supplementary figures and images for: Gut microbiota profiles in diarrheic patients with co-occurrence of Clostridioides difficile and Blastocystis
Source: PLoS One. 2021 Mar 16;16(3):e0248185. doi: 10.1371/journal.pone.0248185 (PMC7963057; doi:10.1371/journal.pone.0248185)

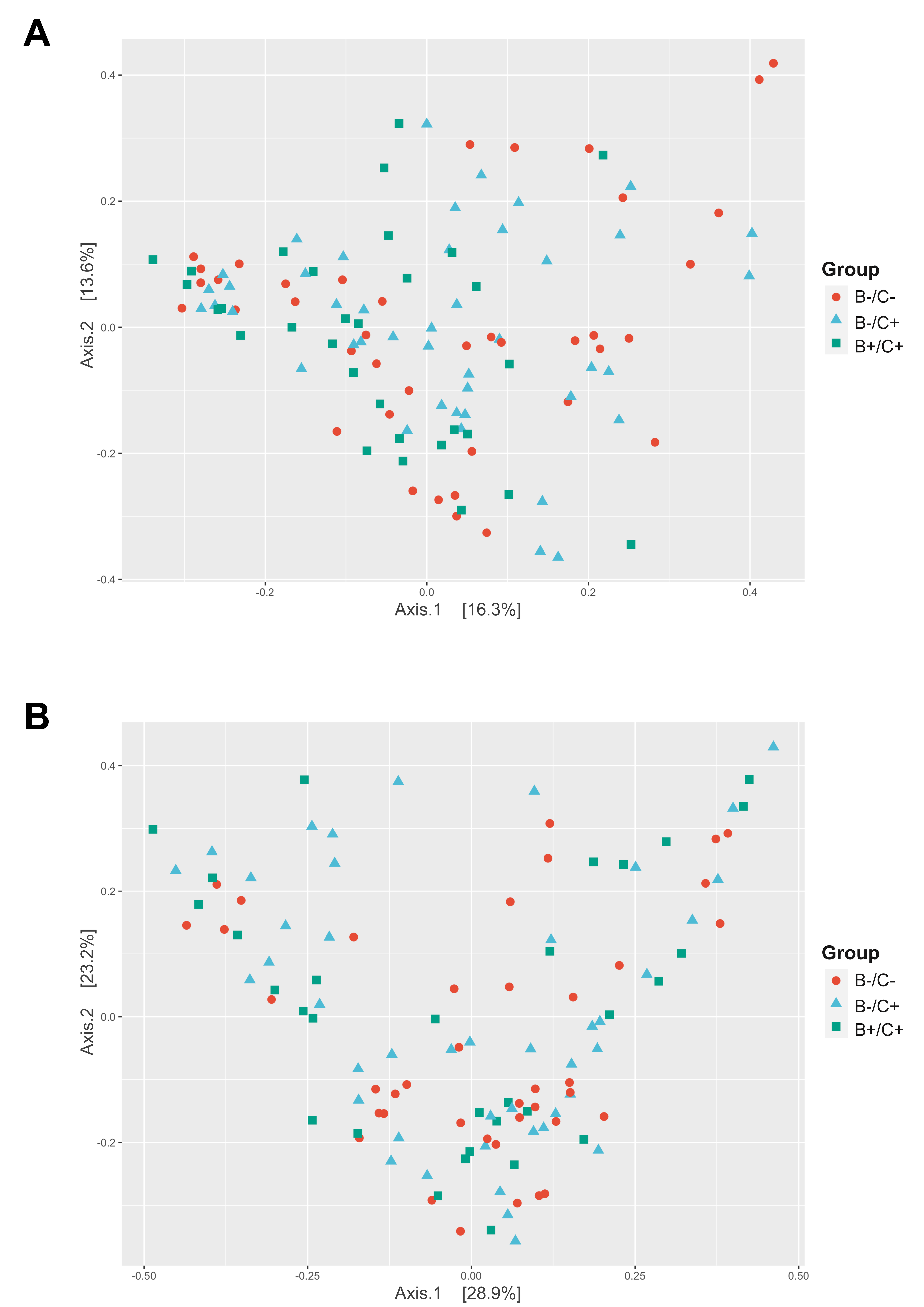

Supplement: S1 Fig — (A) Principal coordinate analysis (PCoA) of the bacterial ASVs. (B) Principal coordinate analysis (PCoA) of the eukaryotic ASVs of the three study groups. The percentage of variation explained by the two dimensions of the PCoA is displayed on the axis. (TIF) [file pone.0248185.s005.tif]

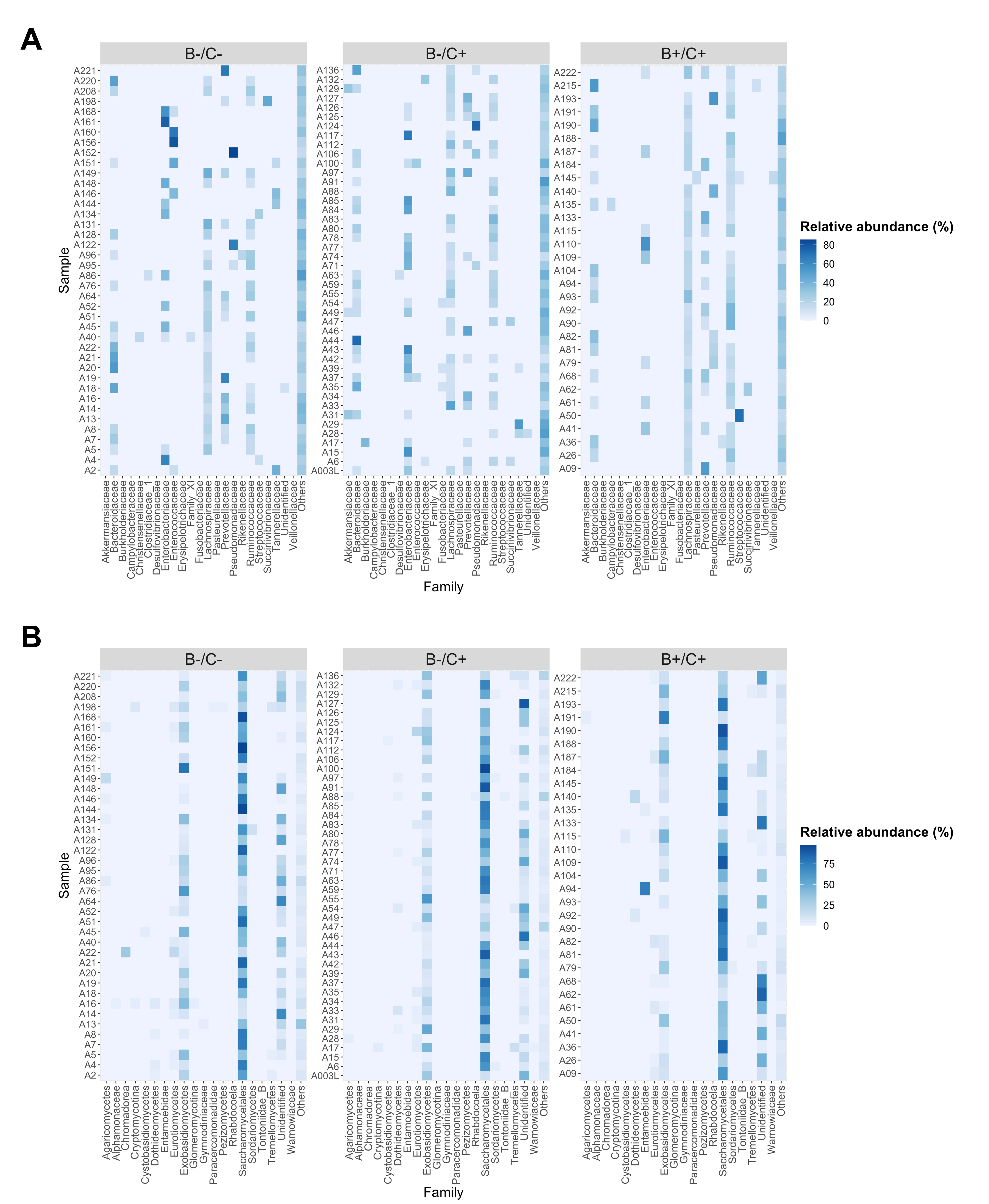

Supplement: S2 Fig — (A) Relative abundance of the 24 most abundant bacterial families of the three study groups. (B) Relative abundance of the 21 most abundant eukaryotic families of the three study groups. (TIF) [file pone.0248185.s006.tif]

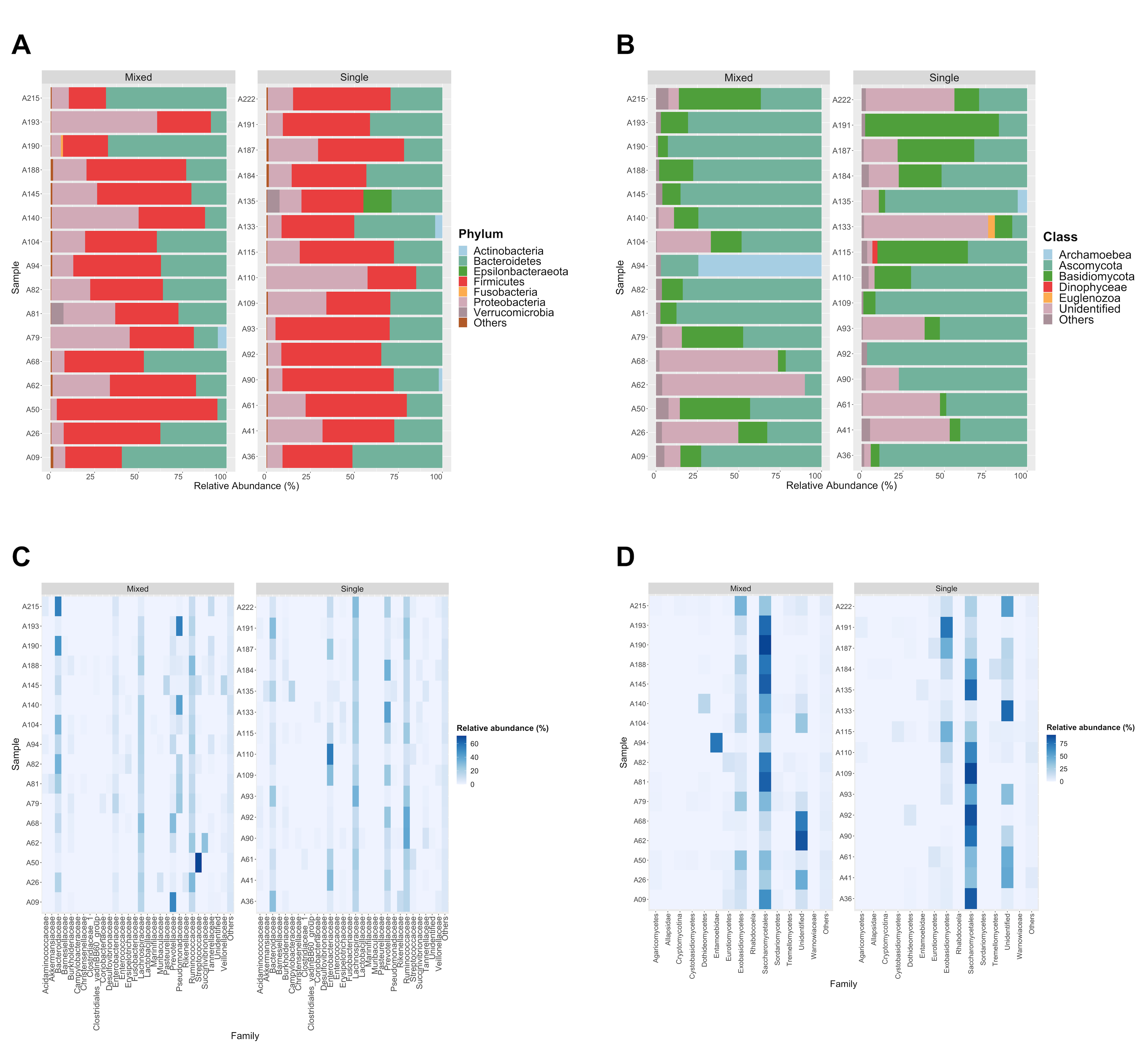

Supplement: S3 Fig — (A) Bar plot of the relative abundance of the bacterial phyla identified in the two subgroups, where Firmicutes, Bacteroidetes, and Proteobacteria are the most abundant. (B) Bar plot of the relative abundance of the eukaryotic classes identified in the two subgroups, where Ascomycota and Basidiomycota where the most abundant. (C) Heatmap of the relative abundance of the 30 most abundant bacterial families identified in the two subgroups. (D) Heatmap of the relative abundance of the 15 most abundant eukaryotic families identified in the two subgroups. (TIF) [file pone.0248185.s007.tif]

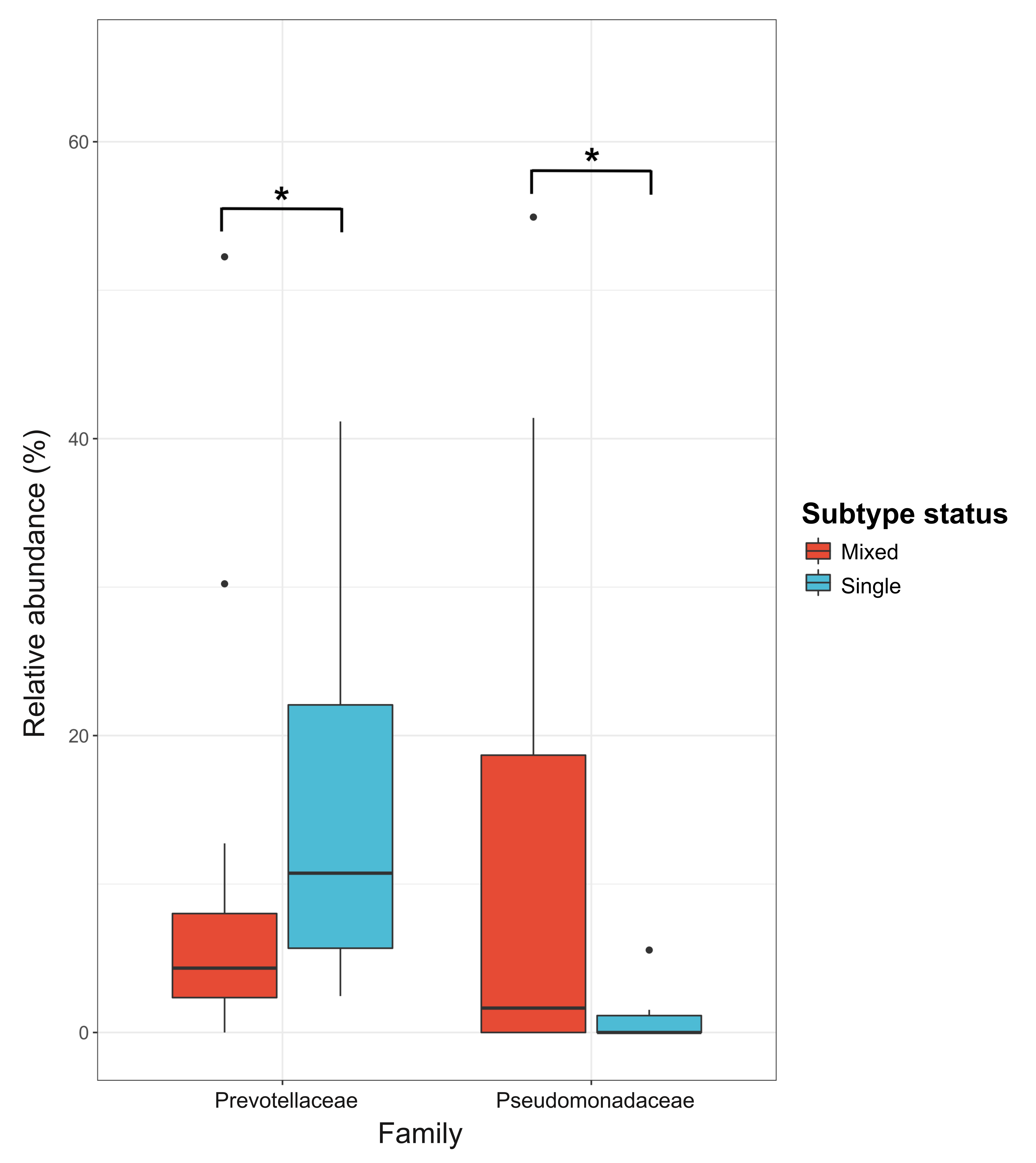

Supplement: S4 Fig — Significant differences between the study groups were evaluated using non-parametric Mann-Whitney test (*, p < 0.05). (TIF) [file pone.0248185.s008.tif]

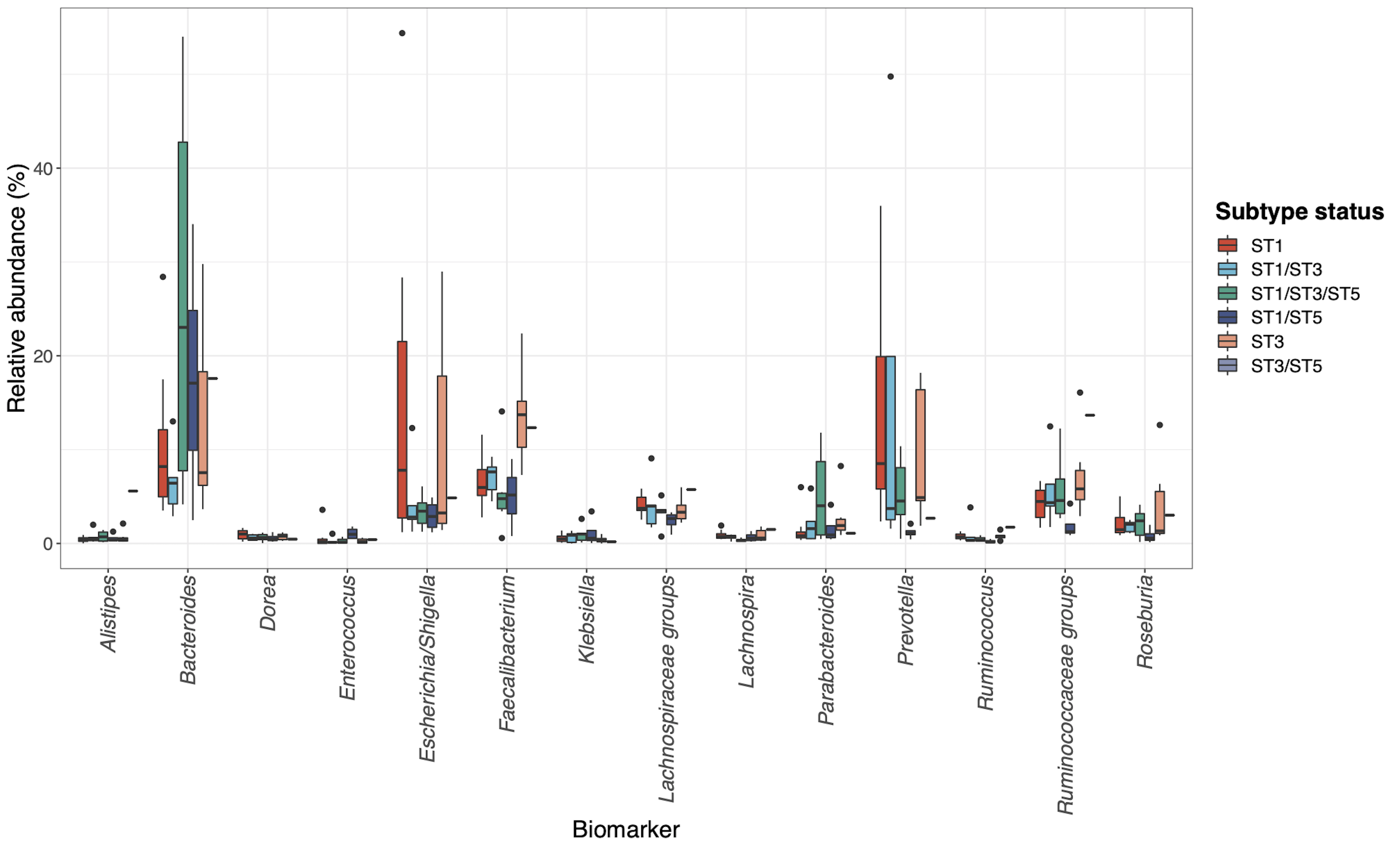

Supplement: S5 Fig — For the boxplot the genera: Acinetobacter, Akkermansia, Bifidobacterium, Bilophila, Eubacterium, Fusobacterium and Methanobrevibacter were not displayed since their relative abundance was low. (TIF) [file pone.0248185.s009.tif]

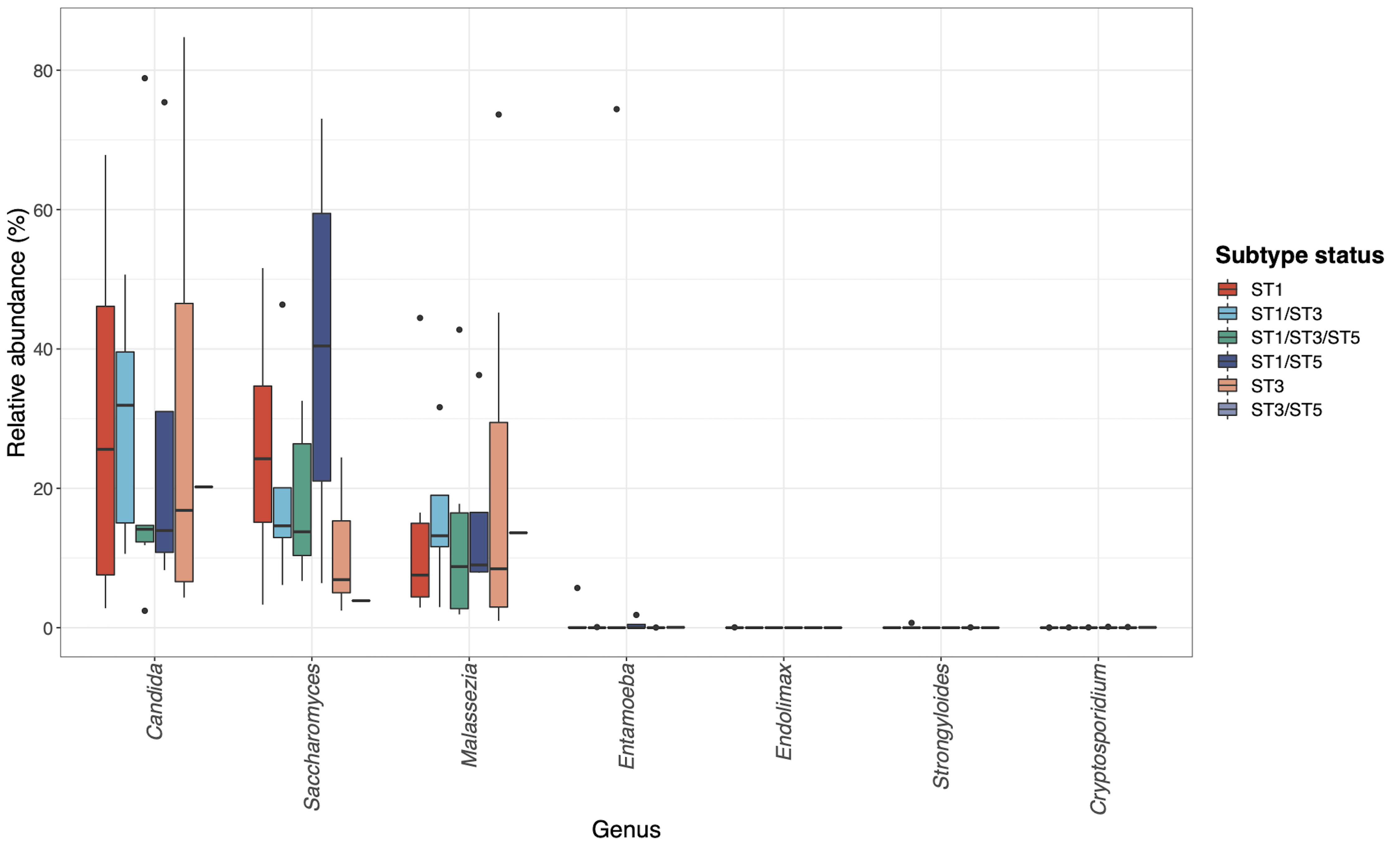

Supplement: S6 Fig — (TIF) [file pone.0248185.s010.tif]
